# A first‐in‐human phase I, multicenter, open‐label, dose‐escalation study of the oral RAF/VEGFR‐2 inhibitor (RAF265) in locally advanced or metastatic melanoma independent from BRAF mutation status

**DOI:** 10.1002/cam4.1140

**Published:** 2017-07-18

**Authors:** Benjamin Izar, William Sharfman, F. Stephen Hodi, Donald Lawrence, Keith T. Flaherty, Ravi Amaravadi, Kevin B. Kim, Igor Puzanov, Jeffrey Sosman, Reinhard Dummer, Simone M. Goldinger, Lyhping Lam, Shefali Kakar, Zhongwen Tang, Oliver Krieter, David F. McDermott, Michael B. Atkins

**Affiliations:** ^1^ Beth Israel Deaconess Medical Center Boston Massachusetts; ^2^ Center for Cancer Precision Medicine/Dana‐Farber Cancer Institute and the Broad Institute Boston Massachusetts; ^3^ Broad Institute of MIT and Harvard Cambridge Massachusetts; ^4^ Sidney Kimmel Comprehensive Cancer Center at Johns Hopkins Baltimore Maryland; ^5^ Dana Farber Cancer Institute Boston Massachusetts; ^6^ Massachusetts General Hospital Boston Massachusetts; ^7^ Abramson Cancer Center of the University of Pennsylvania Philadelphia Pennsylvania; ^8^ California Pacific Medical Center Research Institute San Francisco California; ^9^ Vanderbilt‐Ingram Cancer Center Vanderbilt Tennessee; ^10^ University Hospital Zurich Switzerland; ^11^ Novartis Institutes for BioMedical Research Inc. Cambridge Massachusetts; ^12^ Novartis Pharmaceuticals Corporation East Hanover New Jersey; ^13^ Novartis Pharma AG Basel Switzerland; ^14^ Georgetown‐Lombardi Comprehensive Cancer Center Washington District of Columbia

**Keywords:** Biomarker analysis, BRAF wild‐type, BRAF‐mutant, metastatic melanoma, RAF265

## Abstract

To establish the maximum tolerated dose (MTD), dose‐limiting toxicities (DLT), safety profile, and anti‐tumor efficacy of RAF265. We conducted a multicenter, open‐label, phase‐I, dose‐escalation trial of RAF265, an orally available RAF kinase/VEGFR‐2 inhibitor, in patients with advanced or metastatic melanoma. Pharmacokinetic (PK) analysis, pharmacodynamics (PD) and tumor response assessment were conducted. We evaluated metabolic tumor response by 18[F]‐fluorodeoxyglucose‐positron‐emission tomography (FDG‐PET), tissue biomarkers using immunohistochemistry (IHC), and modulators of angiogenesis. RAF265 has a serum half‐life of approximately 200 h. The MTD was 48 mg once daily given continuously. Among 77 patients, most common treatment‐related adverse effects were fatigue (52%), diarrhea (34%), weight loss (31%) and vitreous floaters (27%). Eight of 66 evaluable patients (12.1%) had an objective response, including seven partial and one complete response. Responses occurred in BRAF‐mutant and BRAF wild‐type (WT) patients. Twelve of 58 (20.7%) evaluable patients had a partial metabolic response. On‐treatment versus pretreatment IHC staining in 23 patients showed dose‐dependent p‐ERK inhibition. We observed a significant temporal increase in placental growth factor levels and decrease in soluble vascular endothelial growth factor receptor 2 (sVEGFR‐2) levels in all dose levels. RAF265 is an oral RAF/VEGFR‐2 inhibitor that produced antitumor responses, metabolic responses, and modulated angiogenic growth factor levels. Antitumor activity occurred in patients with BRAF‐mutant and BRAF‐WT disease. Despite low activity at tolerable doses, this study provides a framework for the development of pan‐RAF inhibitors and modulators of angiogenesis for the treatment of melanoma.

## Introduction

Melanoma remains the most common cause for skin‐cancer related mortality with over 10,000 deaths anticipated in 2016 1. The incidence of melanoma has been steadily rising over the last several decades 1. At the time this trial was launched in 2006, patients with metastatic melanoma had a poor 5‐year survival rate of less than 10% with a median survival of only 6–9 months 2. The poor survival of patients with advanced or metastatic melanoma was primarily due to insensitivity to cytotoxic chemotherapy agents, such as dacarbazine (DTIC). High‐dose interleukin 2 (HD‐IL2) produces durable responses in 5–8% of patients with an overall response rate of 16–22% 3, 4, but the majority of patients were either not suitable for or did not have access to this therapy.

The discovery of somatic activating mutations in the *BRAF* gene in several cancers 5, including approximately 50% of melanomas 6, 7, provided the genetic foundation for the development of targeted treatment approaches for patients with BRAF‐mutant cancers. Activating mutations at V600 codon of the *BRAF* gene, most commonly *BRAF* ^*V600E*^, result in dramatically enhanced activity of the BRAF kinase and constitutive activation of the MAP kinase (MAPK) pathway enabling tumor growth. Targeting the MAP kinase pathway in patients with advanced melanoma was first attempted with sorafenib, either alone or in combination with chemotherapy with ultimately disappointing results 8, 9, 10, making it clear that more selective inhibitors of BRAF were needed.

RAF265 is a novel, orally active, small molecule kinase inhibitor with potent activity against mutant and wild‐type BRAF, CRAF and additional anti‐angiogenic activity through inhibition of vascular endothelial growth factor type 2 (VEGFR‐2) in preclinical models 11, 12, 13. Pan‐RAF inhibitors show activity in preclinical models including those using BRAF‐mutant and BRAF‐WT melanomas 14. Inhibition of VEGFR‐2 using axitinib also showed activity in a phase II trial in patients with metastatic melanoma 15 and the anti‐VEGF antibody bevacizumab enhanced the activity of cytotoxic chemotherapy in a randomized phase II trial 16, particularly in patients with M1c disease especially with elevated LDH. Based on the critical need for effective new treatment approaches for patients with BRAF‐mutant and BRAF‐WT locally advanced or metastatic melanoma, we conducted this first‐in‐human phase I study of RAF265 in this treatment population.

## Patients and Methods

### Patients

Patients with histologically confirmed melanoma; measurable locally advanced or metastatic disease; age ≥ 18 years; an Eastern Cooperative Oncology Group performance status of 0 or 1; available tumor to determine BRAF mutation status; no prior treatment with MEK, VEGF/VEGFR, or RAF inhibitors; no history or presence of brain metastases; a minimum of 4 weeks elapsed since any major surgery or prior anticancer/investigational therapy; and adequate bone marrow, liver and renal function were eligible. Patients signed an institutional review board–approved informed consent form.

### Study design

This study was an open‐label, multicenter, nonrandomized, phase I/II, dose‐escalation study of orally administered RAF265. The study was designed primarily to determine the maximum tolerated dose (MTD) and/or or recommended phase II dose (RP2D), dose‐limiting toxicities (DLTs), and safety of oral dosing of RAF265 in patients with locally advanced or metastatic melanoma using different dosing schedules. The study was comprised of two phases: A dose escalation phase and an expansion phase at the MTD/RP2D. The dose‐escalation phase was guided by pharmacokinetics (PK), pharmacodynamics (PD) and safety. In the dose escalation phase, cohorts 1‐7.1 were administered RAF265 as a single oral dose on Day 1 of the PK run‐in period followed by one loading dose of RAF265 on Cycle 1 Day 1, and by lower once daily doses of RAF265 starting on Cycle 1 Day 2. In the expansion phase (cohort 7.1) the PK run in was omitted and patients received a loading dose in 3 divided doses followed by lower daily dose beginning on Cycle 1, Day 2. The cycle length was 28 days in all cohorts. Here, we report the dose escalation and expansion arm investigating a continuous treatment regimen.

### Study approach

Medical history and demographics were collected at screening. The safety of study medication was evaluated on the basis of rate, type, severity graded in accordance with NCI CTCAE version 3.0. Safety assessments consisted of collecting all AEs, serious adverse events (SAEs) with their severity and relationship with study drug, and pregnancies. They included the regular monitoring of hematology, blood chemistry and urine performed at the study center/central laboratory and regular assessments of vital signs, physical condition, body weight, performance status, and cardiac data (i.e. electrocardiogram (ECG)/echocardiogram (ECHO)). Abnormalities in clinical laboratory tests that were considered clinically significant by the Investigator were recorded on the AE CRF. Dose‐limiting toxicities (DLTs) were defined for various organ systems and other clinically relevant adverse effects (Table S1). The DLT window was the first cycle while AEs were captured for the entire course of treatment. Evaluation of tumor response was conducted at Baseline, and at the end of every 8 weeks of treatment. Response was assessed using the Response Evaluation Criteria in Solid Tumors (RECIST) 1.0. A complete or partial response (CR or PR) was confirmed at least 28 days after the first assessment that documented response and every two cycles thereafter until disease progression, initiation of confounding anticancer therapy, death, loss to follow‐up, withdrawal of consent, or study termination. All patients discontinuing from the study for progressive disease were to have their disease progression documented by radiologic evaluation.

### Pharmacokinetics

Blood samples for plasma RAF265 concentration determination were collected on cycle 1, day 1 and day 15, and on cycle 2, day 1. All PK analyses were based on concentrations of unchanged RAF265 in plasma. PK parameters included the maximum observed concentration (*C*_max_), time to reach *C*_max_ (*T*_max_), area under the curve from time 0 to last measurable concentration (AUC_last_), time point of the last measurable concentration (*T*_last_), and terminal half‐life of elimination phase (*T*_1/2_). Cycle 1 Day 1 and day 15, reported PK parameters include *C*_max_, *T*_max_, AUC_last_ and *T*_last_. Following log‐transformation, the PK parameters AUC and *C*_max_ from the PK run‐in were analyzed separately using a mixed effects model fitting terms for dose and patient (Patient treated as a random factor)‐ln(PK parameter) = a + b*ln(Dose)+error. In this model, the dose‐exposure relationship is mainly characterized by the slope parameter b. A slope lower (higher) than 1 indicates an under (over) proportional dose‐exposure relationship. The point estimate and associated 90% CI for the slope parameter were determined for safety considerations.

### Pharmacodynamics and biomarker assessment

Pharmacodynamic markers were examined using peripheral blood samples, tumor and nevi biopsies (when available) and tumor imaging. BRAF mutation status was determined by Sanger sequencing. Pharmacodynamic markers analyzed, in available tumor/nevus samples obtained before treatment or archival tissue and during treatment on cycle 1, day 8 or day 15, including cytoplasmic p‐MEK, p‐ERK, BIM, c‐KIT, p‐AKT473, pS6 and PTEN, and nuclear p‐ERK, Ki‐67, PARP, cyclin D1, MITF, p27, and p53. Biomarker expression was determined by calculating the percentage of change in the H‐score (14) between on‐treatment and fresh/archival pretreatment specimens. Blood levels of VEGFR‐2 and were assessed before and at multiple time points while on treatment using enzyme‐linked immunosorbent assay (ELISA). Tumor imaging using 18[F]‐FDG‐PET to determine effects of RAF265 on tumor metabolic activity was performed within 2 weeks prior to treatment start (baseline), and on‐treatment on C1D15 and C1D28. Changes in standard uptake value (SUV) were determined by a blinded, independent central imaging review panel. Complete metabolic response (CMR) was complete resolution of tumor FDG‐PET uptake so SUV is the same as background. Partial metabolic response (PMR) decrease in sum of the tumor SUV of ≥25% from the baseline scan. Progressive metabolic disease (PMD) was defined as an increase in tumor SUV of ≥25% from the baseline scan, or the appearance of new FDG‐PET uptake in metastatic lesions. Stable metabolic disease (SMD) was defined as a change in tumor SUV between the PMR and PMD criteria.

### Statistical analyses

A Bayesian Logistic Model with five‐parameter overdose control methodology 17 was used to calculate loading dose, maintenance dose and dose escalation until the MTD was reached with the goal to enroll at last 6 patients at the MTD. The MTD was defined as the dose level with 16–33.3% probability of a DLT. Using the statistical approach the study had a 13.8% chance of identifying the true MTD at DL6. Descriptive statistic were used for the analysis of PK, biomarker analysis and tumor response data.

## Results

### Patient characteristics and study drug exposure

A total of 77 patients were enrolled in this study at four sites in the US and one site in Europe, and received one or more doses of RAF265. Patient demographics and baseline characteristics are listed in Table 1. Among 77 patients, 39 (51%) had BRAF‐mutant and 33 (43%) had BRAF wild‐type melanoma. The BRAF mutation status was unknown for five patients. Sixty five (85.5%) patients had at least one prior line of treatment, and 43 (57%) had at least 2 prior lines of treatment before enrolling in this trial. Patients were enrolled in 8 cohorts corresponding to different dose levels (1 through 7 [Table S2] with a 7–10 days run‐in period, followed by a loading dose (LD) and a daily maintenance dose (MD). Cohort 7.1 omitted the run‐in period and administered the LD in 3 divided doses over 24 h and then received the same daily maintenance dose as in Cohort 7. Twenty‐six patients (40%) received 4 or more cycles and 13 patients (16.9%) received 7 or more cycles of RAF265 with a mean exposure time of 6.1 (±12) months.

**Table 1:** Demographics, clinical data and BRAF mutation status of patients included in this study

Demographic/variable	*N*, (%)
Total population	77 (100%)
Age (years)	
Median	60
Min‐max	26–83
Sex	
Male	43 (55.8)
Female	34 (44.2)
BRAF mutation status	
Mutated	39 (50.6)
Wild‐type	33 (42.9)
Unknown	5 (6.5)
ECOG performance status n (%)	
Grade 0–1 breakdown into 0 and 1?	77 (100)
Prior lines of treatment (n)	
0	11 (14.5)
1	22 (28.9)
2	16 (21.1)
3	27 (35.5)
N/A	1 (1.3)
Stage	
III or IIIC	6 (7.8)
IV	40 (51.9)
IV a	3 (3.9)
IV b	9 (11.7)
IV c	19 (24.7)

### Safety

Treatment–related AEs that occurred in ≥10% of patients are listed in Table 2. The most commonly reported AEs (≥30% of patients) were fatigue (51.9%), diarrhea (35.1%) and weight loss (31.2%). Seven dose‐limiting toxicities occurred in 6 patients, including a grade 3 lipase elevation and grade 2 toxic retinopathy at dose level 6 (48 mg MD) and a grade 3 ataxia and vitreous floaters in one patient, grade 3 diarrhea, and two grade 4 pulmonary emboli at dose levels 7 and 8 (67 mg MD). A total of 30/77 (39%) reported AEs requiring dose adjustment or interruption, most commonly fatigue in 6 patients (7.8%), diarrhea in 5 (6.5%) patients, photopsia, vitreous floaters, hypertension, decreased appetite and nausea in 4 (5.2%) patients each, and thrombocytopenia in 3 patients (3.9%). Based on initial results of 9 patients in dose level 7, a dose expansion cohort was added at 67 mg QD (without initial run‐in period). The overall rate of grade 3 and 4 toxicities was 53.2%. The overall rate of RAF265 related grade 3 and 4 toxicities in dose level 7 and 7.1, including 7 of 19 (36.8%) patients with thrombocytopenia and 4 (21%) patients with diarrhea was unacceptable (Table 3). Specific serious adverse events (SAEs) occurred in 29/77 (37.7%) patients, most commonly (in ≥2 patients) pulmonary embolism in 5 (6.5%) followed by dehydration in 4 (5.2%), thrombocytopenia in 3 (3.9%), and constipation, and retinopathy in 2 (2.6%) patients each. We observed delayed and prolonged, post cycle 1 toxicities in dose level 7 and 7.1. The prior probability of DLTs in these cohorts was below the threshold maximum at the time the final number of patients was enrolled. As described previously 17, post cycle 1 toxicities were therefore used to inform the Bayesian model used in this study, and the maximum‐tolerated dose (MTD) was declared at 48 mg QD (dose level 6). There were 5 (6.5%) deaths reported within 28 days of last administration of the study drug, none of which were deemed to be treatment related.

**Table 2:** Frequency of treatment‐related adverse events across dose levels

Preferred term	DLs 1‐4 *N* = 20 *n* (%)	DL 5 *N* = 15 *n* (%)	DL 6 *N* = 23 *n* (%)	DLs 7 and 7.1 *N* = 19 *n* (%)	All patients *N* = 77 *n* (%)
Fatigue	7 (35.0)	7 (46.7)	13 (56.5)	13 (68.4)	40 (51.9)
Diarrhea	2 (10.0)	3 (20.0)	7 (30.4)	15 (78.9)	27 (35.1)
Weight decreased	2 (10.0)	1 (6.7)	10 (43.5)	11 (57.9)	24 (31.2)
Vitreous floaters	1 (5.0)	3 (20.0)	9 (39.1)	8 (42.1)	21 (27.3)
Dysgeusia	1 (5.0)	1 (6.7)	9 (39.1)	8 (42.1)	19 (24.7)
Nausea	2 (10.0)	1 (6.7)	8 (34.8)	8 (42.1)	19 (24.7.)
Photopsia	1 (5.0)	4 (26.7)	6 (26.1)	8 (42.1)	19 (24.7)
Decreased appetite	0 (0.0)	0 (0.0)	8 (34.8)	9 (47.4)	17 (22.1)
Thrombocytopenia	1 (5.0)	0	4 (17.4)	11 (57.9)	16 (20.8)
Muscle spasms	2 (10.0)	3 (20.0)	5 (21.7)	5 (26.3)	15 (19.5)
Vomiting	2 (10.0)	0	6 (26.1.4)	7 (36.8)	15 (19.5)
Hypertension	3 (15.0)	0	5 (21.7)	4 (21.1)	12 (15.6)
Abdominal pain	1 (5.0)	0	3 (13.0)	3 (15.8)	7 (9.1)
Constipation	1 (5.0)	1 (6.7)	2 (8.7)	3 (15.8)	7 (9.1)
Lipase increased	1 (5.0)	2 (13.3)	4 (17.4)	2 (10.5)	7 (9.1)
Dizziness	1 (5.0)	1 (6.7)	4 (17.4)	3 (15.8)	9 (11.7)
Rash	0	1 (6.7)	4 (17.4)	1 (5.3)	6 (7.8)
Neutropenia	0	0	0	5 (26.3)	5 (6.5)
Alopecia	2 (10.0)	1 (6.7)	1 (4.3)	0	4 (5.2)
Dehydration	0	0	3 (13.0)	1 (5.3)	4 (5.2)
Dry mouth	0	0	1 (4.3)	3 (15.8)	4 (5.2)
Dyspnea	0	0	2 (8.7)	2 (10.5)	4 (5.2)
Hemoglobin decreased	0	0	2 (8.7)	2 (10.5)	4 (5.2)

**Table 3:** Frequency of treatment‐emergent grade 3/4 events across dose levels

Grade 3/4 toxicities	DLs 1–5 (≤24 mg; *N* = 35) *n* (%)	DL 6 (48 mg; *N* = 23) *n* (%)	DLs 7 and 7.1 (67 mg; *N* = 19) *n* (%)	All DLs *N* = 77 *n* (%)
Total (n)	3	15	26	41 (53.2%)
Hematological	–	1 (4.3%)	11 (57.9)	12 (29.2%)
Thrombocytopenia	–	0	7 (36.8)	7 (17.1%)
Neutropenia	–	1 (4.3%)	3 (15.8)	4 (9.8%)
Pancytopenia			1 (5.3)	1 (2.4%)
Fatigue	–	3 (13)	3 (15.8)	6 (14.4%)
Diarrhea	–	–	4 (21.1)	4 (9.8%)
Hypertension	–	2 (8.7)	2 (10.5)	4 (9.8%)
Vitreous floaters	–	1 (4.3)	1 (5.3)	2 (4.8%)
Weight loss	–	1 (4.3)	–	1 (2.4%)
Decreased appetite	–	–	2 (10.5)	2 (4.8%)
Pulmonary embolism	–	1 (4.3)	1 (5.3)	2 (4.8%)
Dyspnea	–	1 (4.3)	–	1 (2.4%)
Angina unstable	–	–	1 (5.3)	1 (2.4%)
Ataxia	–	–	1 (5.3)	1 (2.4%)
Lipase increased		2 (8.7)	–	2 (4.8%)
Abdominal pain		1 (4.3)		1 (2.4%)
Blood lactic acid increased		1 (4.3)		1 (2.4%)
Hypophosphatemia	1 (2.9)			1 (2.4%)
Squamous cell carcinoma	1 (2.9)	1 (4.3)		2 (4.8%)
Basal cell carcinoma	1 (2.9)			1 (2.4%)
Cutaneous lupus erythematosus			1 (5.3)	1 (2.4%)

### Pharmacokinetics

RAF265 had an extensive distribution phase and a long half‐life of approximately 200 h or 8 days (Table S3). Preclinical studies indicated efficacious concentration (*C*_eff_) was 4127 ng/mL, which corresponded to estimated plasma *C*_eff_ between MTD of 48 mg (dose level 6) and 67 mg (dose level 7/7.1). In general, the mean observed maximum plasma concentration (*C*_max_) and area under the curve (AUC) increased nearly proportionally with dose.

### Antitumor efficacy

A total of 66 patients across all dose levels were evaluable for antitumor response to RAF265. Among these, there were a total of eight RECIST defined responses resulting in an objective response rate (ORR) of 12.1% across all dose levels (Table 4). These included seven partial responses (PR) and one complete response (CR). Four PR were observed in 39 patients with *BRAF* ^*V600E*^ mutation and two PR and one CR in 33 patients with BRAF‐WT melanomas and one PR in a patient with an unknown BRAF mutation status (Table 4). The median duration of response was 18.3 months (range, 1.4–51.7 months) in responders.

**Table 4:** Antitumor response rates based on dose levels (DL) and BRAF mutation status

	DLs 1 to 4 *N* = 20 *n* (%)	DL 5 *N* = 15 *n* (%)	DL 6 *N* = 23 *n* (%)	DLs 7 and 7.1 *N* = 19 *n* (%)	All patients *N* = 77 *n* (%)
Best overall response					
BRAF mutation					
Evaluable disease	6	8	10	9	33
Complete response (CR)	0	0	0	0	0
Partial response (PR)	2 (33.3)	1 (12.5)	1 (10)	0	4 (12)
Stable disease (SD)	0	3 (37.5)	7 (70)	5 (56)	15 (46)
Progressive disease (PD)	4 (66.7)	4 (50)	2 (20)	4 (44)	14 (42)
BRAF wild‐type					
Evaluable disease	8	5	8	7	28
Complete response (CR)	0	0	0	1 (14.3)	1 (3.6)
Partial response (PR)	1 (12.5)	0	1 (12.5)	0	2 (8)
Stable disease (SD)	1 (12.5)	3 (60)	2 (25)	5 (71.4)	11 (39.3)
Progressive disease (PD)	6 (75)	2 (40)	5 (62.5)	1 (14.3)	14 (50)
BRAF unspecified/unknown					
Evaluable disease	1	0	2	2	5
Complete response (CR)	0	0	0	0	0
Partial response (PR)	0	0	0	1 (50)	1 (20)
Stable disease (SD)	1 (100)	0	0	0	1 (20)
Progressive disease (PD)	0	0	2 (100)	1 (50)	3 (60)
Total					
Evaluable disease	15	13	20	18	66
Complete response (CR)	0	0	0	1 (5.6)	1 (1.5)
Partial response (PR)	3 (20)	1 (7.7)	2 (10)	1 (5.6)	7 (10.6)
Stable disease (SD)	2 (13.3)	6 (46.1)	9 (45)	10 (55.6)	27 (40.9)
Progressive disease (PD)	10 (66.7)	6 (46.1)	9 (45)	6 (33.3)	31 (47)
Best response					
CR or PR	3 (20)	1 (7.7)	2 (10)	2 (11.1)	8 (12.1)
CR, PR or SD	5 (33.3)	7 (53.8)	11 (55)	12 (66.7)	35 (53)
SD or better after 12 months	3 (20)	1 (7.7)	2 (10)	1 (5.6)	7 (10.6)

### Pharmacodynamic and biomarker analyses

**Figure 1:**
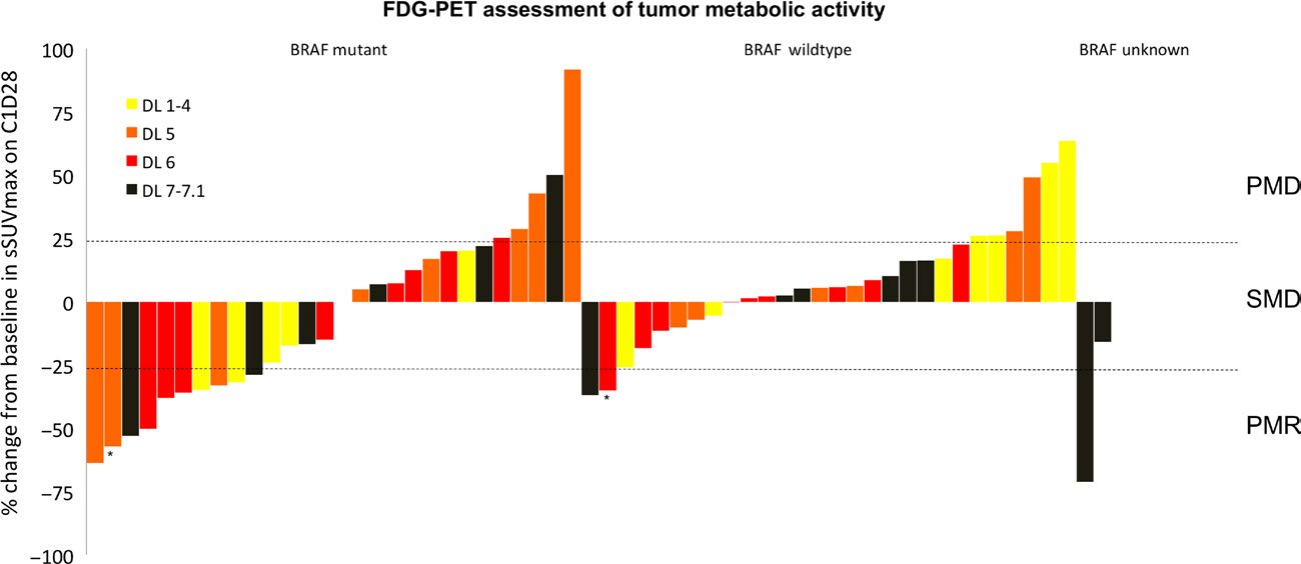
Metabolic responses based on FDG‐PET assessment. PMR partial metabolic response, SMD stable metabolic disease, PMD progressive metabolic disease. ^*^indicates patients with PMD as defined in Methods.

**Figure 2:**
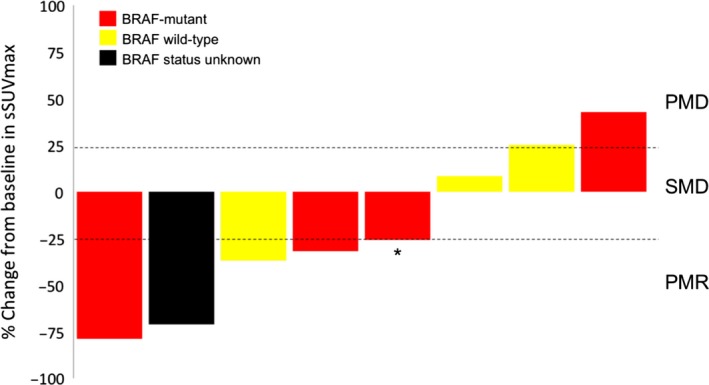
Relationship between RECIST‐defined responders and metabolic responses. ^*^indicates a patient with RECIST‐defined CR.

The effect of RAF265 on tumor metabolic activity was assessed by tumor imaging using 18[F]‐FDG‐PET. Baseline and evaluable C1D15 and C1D28 FDG‐PET scans were available in 71, 65 and 58 patients, respectively. Across all dose levels, 12 of 58 (20.7%) patients exhibited a partial metabolic response (PMR) (Fig. 1), 36 (62.1%) had stable metabolic disease (SMD) and 10 (17.2%) had progressive metabolic disease (PMD) on C1D28. Across all dose levels, 10 of 65 (15.4%) patients had PMR, 46 (70.8%) had SMD and 9 (13.8%) had PMD on C1D15. PMR occurred in 8 of 28 (28.6%) patients with BRAF‐mutant tumors, and 3 of 28 (10.7%) patients with BRAF‐WT disease on C1D28. On C1D15 imaging, we observed PMR in 8 of 33 (24.2%) patients with BRAF mutant tumors, and 1 of 29 (3.4%) patients with BRAF WT disease. Responses occurred statistically more significant in patients with BRAF‐mutant disease on C1D15 (*P* = 0.028; two‐tailed Fisher's test) and there was a trend toward higher metabolic responses in these patients on C1D28 (*P* = 0.1808; two‐tailed Fisher's test). Five of eight patients with RECIST‐defined responses also had partial metabolic response (Fig. 2).

In 23 patients, on‐treatment biopsies were available for IHC analysis to assess for changes in biomarker expression compared to pretreatment biopsies (Tables S4 and S5). Across all dose levels, there was variability in expression changes, including significant increases and decreases in marker expression for the examined proteins and phospho‐proteins. Cytoplasmic and nuclear p‐ERK was modestly decreased in the on‐treatment specimens of patients in DL6‐7.1, with median decrease in 13.4–50% and 7.6–22.2%, respectively. The median cytoplasmic p‐ERK abundance was increased by 27–52.5% in patients treated with DL1‐5. Abundance of p‐MEK and BIM were modestly changed. Interestingly, a dose‐dependent increase in p27 abundance (median increase 30.4% and 72.2% in DL 6 and DL 7/7.1, respectively), and decrease in cyclin D1 (median decrease 25%) and Ki67 (up to 51.8% in DL 6) were seen, together indicating a decreased rate of entry into S‐phase and proliferation. Although overall levels of p‐AKT and pS6 showed only modest changes, both were decreased in DL 6 (median decrease 46.7% and 35.8%, respectively).

**Figure 3:**
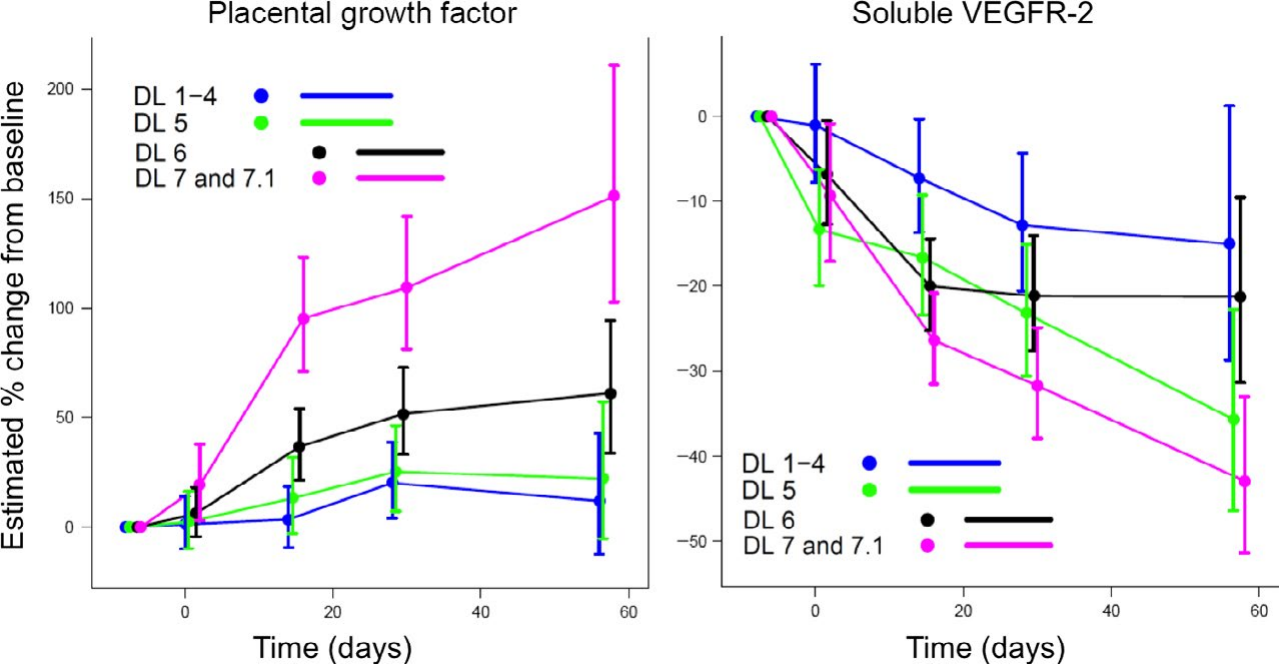
Relative protein abundance of placental growth factor (PGIF) and soluble VEGFR‐2 (sVEGFR‐2) measured in blood across dose levels.

Statistically significant, dose‐dependent changes in the modulation of the VEGF pathway across all dose levels over time were seen (Fig. 3). After 60 days of treatment, soluble VEGFR‐2 levels decreased by 15–42% from baseline. In contrast, protein levels of placental growth factor increased by 50–150% in patients treated at dose levels 6, 7 and 7.1.

## Discussion

Here, we report the first in‐human experience of the orally available, RAF/VEGFR‐2 inhibitor RAF265 in patients with locally advanced or metastatic melanoma. Overall, RAF265 is an agent with a long serum half‐life of ~8 days, and a MTD of 48 mg daily. Doses above 48 mg daily were associated with unacceptable acute, delayed and prolonged toxicity. Across the evaluable study population, the antitumor response rate was 12.1%. Notably, these responses occurred in patients with tumors with or without *BRAF* mutation and across dose levels. Furthermore, metabolic responses were seen in 20.7% of patients, and significant alterations of placental growth factor and sVEGFR‐2, both key modulators of angiogenesis, were also observed.

The discovery of sensitizing BRAF mutations in melanoma provided a strong biologic rationale for the development and the rapid implementation of BRAF‐inhibitors into clinical trials. Preclinical studies showed that RAF265 selectively inhibited *BRAF* ^*V600E*^, CRAF, VEGFR‐2 and other targets at nanomolar EC50 concentrations 11, 12, 13. This data suggested that RAF265 might produce superior results to those seen with sorafenib in patients with BRAF‐mutant melanoma. While RAF265 showed a relatively low response rate and some significant and prolonged toxicities (particularly thrombocytopenia and visual problems) making its therapeutic index suboptimal for further pursuit, attempting to selectively target mutant BRAF proved to be a sound strategy as evident by the activity observed with more selective BRAF inhibitors such as vemurafenib and dabrafenib in this patient population. Specifically vemurafenib or dabrafenib either as single agents or in combination with the MEK‐inhibitors cobimetinib or trametinib, produced objective response rates between 50% and 70%, and improved median progression‐free and overall survival in patients with *BRAF*‐mutant advanced or metastatic melanoma, leading to their FDA‐approvals 18, 19, 20, 21, 22.

Preclinical data indicated that RAF265 had a similar an EC50 (=14 nmol/L for *BRAF* ^*V600E*^) as vemurafenib. However, in comparison to vemurafenib, response rates were lower in *BRAF* ^*V600E*^ patients. A distinguishing feature of RAF265 from vemurafenib and dabrafenib is the clinical activity in BRAF‐WT patients. In contrast, RAF265 seems to achieve plasma levels that are commensurate with active exposures in preclinical models; however, the agent may not have sufficient selectivity for *BRAF* ^*V600E*^ in most patients, as indicated by the increased incidence of thrombocytopenia and visual side effects—both more consistent with inhibition of wild‐type BRAF and CRAF, and decreased incidence of skin squamous cell carcinoma. In line with this, we observed an increase in p‐ERK expression in patients treated at DL1‐5, and moderately decreased p‐ERK abundance in patients treated at DL 6‐7.1. In comparison, vemurafenib suppresses pERK by well over 80% at the recommended phase II dose 23. Overall, this highlights the need for highly selective inhibitors in order to reduce off‐target effects that result in toxicities, which collaterally limit tolerability of potentially clinical beneficial drugs, as seen with, for example, sorafenib in the treatment of either melanoma or renal cell cancer relative to either more selective BRAFV600 inhibitors (e.g. vemurafenib) for melanoma 24 or VEGF inhibitors (e.g., axitinib) in renal cell cancer 25.

We observed responses in patients with BRAF‐WT melanoma, including a complete response. This is consistent with preclinical observations of responses in BRAF‐WT patient‐derived xenografts treated with RAF265 12 and other pan‐RAF‐inhibitors 14. Given RAF265′s relatively high EC50 (>5 *μ*mol/L) for the wild‐type BRAF protein, it is conceivable that these responses were not due to BRAF inhibition. Inhibition of wild‐type BRAF with vemurafenib, dabrafenib and related BRAF inhibitors results in transactivation of alternative RAF‐dimers and downstream activation of ERK 26. However, RAF265 appears to inhibit MAPK pathway signaling in some RAS‐mutant models 12. It is plausible that responses in BRAF‐WT patients stem from inhibition of other targets highlighting the pan‐RAF inhibitor/multi‐kinase inhibitor function of RAF265. These effects may be explained by inhibition of the MAPK pathway given that MEK inhibitors are associated with responses in BRAF WT melanoma 27, 28, and other cell autonomous or cell non‐autonomous mechanisms, which is supported by our biomarker analysis. First, our biomarker analysis indicates that RAF265 also interferes with the AKT pathway, S‐phase entry and cell proliferation, supporting properties of a multi‐kinase inhibitor. In support of the latter, we observed a significant decrease in sVEGFR‐2 levels over time across all dose levels. At the same time, we observed increased levels of placental growth factor, one of the VEGFR‐1 ligands, emphasizing the modulatory effects of RAF265 on angiogenesis. Clinically this is also reflected the development of hypertension in 17% of patients (Table 2), a common side effect of anti‐VEGF directed therapy. Previous studies, including a phase II trial with the VEGFR1‐3 inhibitor axitinib, demonstrated a ~19% response rate in patients with metastatic melanoma 15. Furthermore, the role of anti‐angiogenic therapies in combination with other treatments, such as immune checkpoint inhibitors or chemotherapy, is currently under study as a treatment option for patients with metastatic melanoma 29.

Responses to RAF265 in BRAF‐WT patients may also be associated with alternative mutations that were not systematically evaluated in this study, such as mutations in NRAS, which occur mutually exclusive from BRAF mutations in ~25% of patients, non‐V600E BRAF mutations in ~5–10% of patients, NF1 in ~10–15% of patients and PTEN loss, which is found in ~10% of melanoma and occur with or without concomitant BRAF mutation 6, 7.

We observed partial metabolic responses in 20.7% and stable metabolic disease in 43% of patients. Previous work found a correlation between metabolic and RECIST‐defined responses in patients with BRAF‐mutant melanoma treated with vemurafenib 30. While our analysis is limited by a small number of patients with both clinical and concomitant metabolic responses, we find that five of eight RAF265‐treated patients with RECIST‐defined response (PR or CR) also had partial metabolic responses (Fig. 2). Changes in glucose metabolism in BRAF‐mutant melanoma have been suggested as physiologic basis of BRAF‐inhibitor associated PET‐CT responses, and may underlie changes identified in this study 30, 31, 32. However, discordant metabolic and RECIST‐response in two patients in our study indicate that other metabolic pathways may be related to RAF/VEGFR‐2 induced antitumor activity.

Treatment options for patients with BRAF WT patients primarily include immune checkpoint inhibitors and HD‐IL2. Immune checkpoint inhibitors, such as ipilimumab, nivolumab and pembrolizumab either as single agents, or nivolumab and ipilimumab in combination, produce durable responses in 10 to over 50% of patients 33, 34, 35, 36, 37, 38. Choosing the most effective treatment for patients with BRAF‐WT melanoma who have not responded or are ineligible for immunotherapy remains a clinical challenge. In this context, our study highlights the necessity to obtain a deeper understanding of genetic predictors for response—beyond BRAF—in order to identify subsets, where therapies such as RAF265 or other pan‐RAF inhibitors might be beneficial.

In conclusion, we have shown that RAF265, an orally available RAF/VEGFR‐2 inhibitor, produces clinical and metabolic responses in a subset of patients with locally advanced or metastatic melanoma, including patients with BRAF‐WT disease. The MTD was determined to be 48 mg PO daily as higher doses produced visual problems and prolong thrombocytopenia. Furthermore, RAF265 significantly modulates key regulators of angiogenesis, including VEGFR‐2 and PGIF. A long half‐life and prolonged toxicities along with a relatively low response rate indicate that RAF265 has limited selectivity or activity for BRAF in vivo. This marginal therapeutic index together with the advent of other much more effective and selective BRAFV600 inhibitors resulted in the phase II dose expansion study being cancelled. Responses in patients with BRAF‐WT melanoma indicate that the development of more specific pan‐RAF inhibitors may provide a feasible therapeutic avenue for these patients. This study also provides rationale to continue ongoing efforts investigating the combination of VEGFR‐2 targeted therapies with immunotherapies.

## Conflict of Interest

W. Sharfman serves as consultant for Merck, Novartis and Castle Bioscience; received research funding from BMS and Merck. F. S. Hodi served as consultant for Novartis. K. T. Flaherty served as consultant for Novartis and receives research funding from Novartis. J. Sosman served on advisory boards for Merck, Genentech and Array. R. Dummer serves as consultant or has advisory board relationship with Novartis, Merck Sharp & Dhome, BMS, Roche, GSK, Amgen; received research funding from Novartis, Merck Sharp & Dhome (MSD), BMS, Roche, GSK. S. M. Goldinger has advisory board relationship and received travel funding from BMS, Novartis and Roche. D. F. McDermott participated at an advisory board for Novartis. M. B. Atkins serves as consultant for Novartis, Genentech, BMS, Merck, Amgen, Pfizer, Astra Zeneca, Nektar, Agenus and Celldex. L. P. Lam, S. Kakar, Z. Tang are employed by Novartis. B. Izar, D. Lawrence, R. Amaravadi, K. B. Kim and I. Puzanov have nothing to disclose.

## Supporting information


**Table S1.** Definition of dose limiting toxicities (DLTs).
**Table S2.** Scheme of eight dose levels. At each dose level, with the exception of DL 8, patients received a 7–10 days run‐in dose, followed by a single loading dose (LD) and a daily maintenance dose (MD). *administered as three doses.
**Table S3.** Pharmacokinetics of RAF265.
**Table S4.** Changes in expression of cytosolic biomarkers. Patients included in this analysis had a fresh or archival tissue scored as described in the methods section. Changes of biomarker abundance were evaluated in on‐treatment specimens. Shown is the number of patients with tissue available for each biomarker analysis, mean and median % changes compared to the pretreatment specimen, and the range for each biomarker in each dose level and in the entire evaluable population. **pMEK, phosphorylated MAPK/ERK kinase; pERK, phosphorylated extracellar signal‐regulated kinase; Ki67, proliferation‐associated antigen Ki‐67; BIM, a pro‐apoptotic member of the BCL‐2 family; PARP, poly(ADP‐ribose)polymerase; Cyclin D1, cell cycle gene, MITF, microphthalmia‐associated transcription factor; CKIT, c‐KIT; P53, tumor protein 53/TP53; PAKT473, phospho Akt S 473; PS6, phosphoserine 240‐S6 ribosomal protein; PTEN, phosphatase and tensin homolog.
**Table S5.** Changes in expression of nuclear biomarkers.
