# Supplementary material for: A first‐in‐human phase I, multicenter, open‐label, dose‐escalation study of the oral RAF/VEGFR‐2 inhibitor (RAF265) in locally advanced or metastatic melanoma independent from BRAF mutation status
Source: Cancer Med. 2017 Jul 18;6(8):1904–14. doi: 10.1002/cam4.1140 (PMC5548886; doi:10.1002/cam4.1140)
Supplement: Supplementary file 1 — Table S1. Definition of dose limiting toxicities (DLTs). Table S2. Scheme of eight dose levels. At each dose level, with the exception of DL 8, patients received a 7–10 days run‐in dose, followed by a single loading dose (LD) and a daily maintenance dose (MD). *administered as three doses. Table S3. Pharmacokinetics of RAF265. Table S4. Changes in expression of cytosolic biomarkers. Patients included in this analysis had a fresh or archival tissue scored as described in the methods section. Changes of biomarker abundance were evaluated in on‐treatment specimens. Shown is the number of patients with tissue available for each biomarker analysis, mean and median % changes compared to the pretreatment specimen, and the range for each biomarker in each dose level and in the entire evaluable population. **pMEK, phosphorylated MAPK/ERK kinase; pERK, phosphorylated extracellar signal‐regulated kinase; Ki67, proliferation‐associated antigen Ki‐67; BIM, a pro‐apoptotic member of the BCL‐2 family; PARP, poly(ADP‐ribose)polymerase; Cyclin D1, cell cycle gene, MITF, microphthalmia‐associated transcription factor; CKIT, c‐KIT; P53, tumor protein 53/TP53; PAKT473, phospho Akt S 473; PS6, phosphoserine 240‐S6 ribosomal protein; PTEN, phosphatase and tensin homolog. Table S5. Changes in expression of nuclear biomarkers. [file CAM4-6-1904-s001.docx]

**Appendix Table 1:** Definition of dose limiting toxicities (DLTs).

**Appendix Table 2:** Scheme of eight dose levels. At each dose level, with the exception of DL 8, patients received a 7-10 day run-in dose, followed by a single loading dose (LD) and a daily maintenance dose (MD). *administered as three doses.

**Appendix Table 3:** Pharmacokinetics of RAF265.

**Appendix Table 4:** Changes in expression of cytosolic biomarkers. Patients included in this analysis had a fresh or archival tissue scored as described in the methods section. Changes of biomarker abundance were evaluated in on-treatment specimens. Shown is the number of patients with tissue available for each biomarker analysis, mean and median % changes compared to the pre-treatment specimen, and the range for each biomarker in each dose level and in the entire evaluable population. ** pMEK = phosphorylated MAPK/ERK kinase, pERK = phosphorylated extracellar signal-regulated kinase, Ki67 = proliferation-associated antigen Ki-67, BIM= a pro-apoptotic member of the BCL-2 family, PARP = Poly(ADP-ribose)polymerase, Cyclin D1 = cell cycle gene, MITF = microphthalmia-associated transcription factor, CKIT=c-KIT, P53= Tumor Protein 53/TP53, PAKT473= Phospho Akt S 473, PS6=Phosphoserine 240-S6 ribosomal protein, PTEN=Phosphatase and Tensin homolog.

**Appendix Table 5:** Changes in expression of nuclear biomarkers.

**Appendix Table 1: Definition of DLTs**

| **TOXICITY** | **ANY OF THE FOLLOWING CRITERIA** |
| --- | --- |
| Hematologic* | CTCAE Grade 4 neutropenia - for > 7 consecutive days |
|  | CTCAE Grade 3 thrombocytopenia - for > 7 consecutive days |
|  | CTCAE Grade 4 thrombocytopenia (confirmed on repeated testing) |
|  | CTCAE ≥Grade 3 neutropenia with fever (i.e., fever > 38.5°C with an ANC < 1000 cells/mm3) |
| Renal | Serum creatinine ≥ 2.0 x ULN to ≤ 3.0 x ULN for > 7 consecutive days |
|  | ≥ CTCAE Grade 3 serum creatinine |
| Hepatic | Total bilirubin ≥ 2.0 x ULN to ≤ 3.0 x ULN for > 7 consecutive days |
|  | ≥ CTCAEGrade 3 bilirubin |
|  | CTCAE Grade 3 AST or ALT for ≥ 7 consecutive days |
|  | CTCAE Grade 4 AST or ALT |
| Serum Amylase/Lipase | Asymptomatic ≥ CTCAE Grade 3 for > consecutive 7 days. |
|  | Pancreatitis CTCAE ≥ Grade 3: abdominal pain with elevation of serum amylase/lipase of any CTCAE grade. |
| Cardiac | Prolonged QTcF ≥ CTCAE Grade 3 |
|  | All other CTCAE ≥ Grade 3 cardiac events. |
| Hypercholesterolemia/Hypertriglyceridemia | CTCAE Grade 4 hypercholesterolemia/ hypertriglyceridemia despite use of appropriate lipid-lowering agent. |
| Hypertension | CTCAE Grade 4 (hypertensive crisis) |
|  | CTCAE Grade 2 or 3 - ONLY IF the diastolic blood pressure does not stabilize to ≤ 20 mmHg (or clinically acceptable range for that patient) of pretreatment (baseline) diastolic blood pressure, despite having used concomitant antihypertensive treatments for ≥ 7 days |
| Neurotoxicity | > 1 CTCAE Grade level increase |
| Ocular/Visual | CTCAE Grade ≥1 retinopathy/retinal detachment  All other CTCAE Grades 3 or 4 |
| Other adverse events* | CTCAE Grade 3 adverse events (excluding CTCAE Grade 3 elevations in alkaline phosphatase) which cause an inability to administer RAF265 for > 14 consecutive days |
|  | CTCAE Grade 4 adverse events (excluding CTCAE grade 4 elevations in alkaline phosphatase) |
|  | ≥ CTCAE Grade 3 vomiting or CTCAE grade 3 nausea despite the use of standard anti-emetics |
|  | ≥ CTCAE Grade 3 diarrhea despite the use of optimal anti-diarrheal treatments |
|  | Any other adverse event unrelated to disease progression, inter-current illness, or concomitant medications that caused an inability to administer RAF265 > 14 days. |
| * Note: ≥ CTCAE Grade 3 anemia will NOT be considered a DLT unless judged to be a hemolytic process secondary to study drug. ≥ CTCAE Grade 3 lymphopenia will NOT be considered a DLT unless clinically significant. | |
| An AE must be clinically significant to define DLT: alopecia, study drug-related fever, electrolyte abnormalities (including K, Na, Cl, HCO_3_, Mg, Ca, PO_4_) that are ≤ Grade 3 will not be considered DLTs unless clinically significant | |

**Appendix Table 2: Dosing scheme of different dose-levels.**

| Dose level | Run-in | LD | MD | N |
| --- | --- | --- | --- | --- |
| 1 | 10 mg | 8 mg | 2 mg | 3 |
| 2 | 20 mg | 16 mg | 3 mg | 3 |
| 3 | 36 mg | 29 mg | 6 mg | 4 |
| 4 | 72 mg | 58 mg | 12 mg | 10 |
| 5 | 144 mg | 115 mg | 24 mg | 15 |
| 6 | 288 mg | 230 mg | 48 mg | 23 |
| 7 | 403 mg | 322 mg | 67 mg | 9 |
| 7.1 | None | 288 mg* | 67 mg | 10 |

**Appendix Table 3: Pharmacokinetics of RAF265**

| **Study**  **day** | **PK parameter** | **DL 1 (N=3)** | **DL 2 (N=3)** | **DL 3 (N=4)** | **DL 4 (N=10)** | **DL 5 (N=15)** | **DL 6 (N=23)** | **DL 7 (N=9)** | **DL 7.1 (N=10)** |  |
| --- | --- | --- | --- | --- | --- | --- | --- | --- | --- | --- |
| First  dose* | **Cmax (ug/mL)** |  |  |  |  |  |  |  |  |  |
|  | n | 3 | 3 | 4 | 10 | 15 | 22 | 9 | 0 |  |
|  | Mean  (SD) | 0.14 (0.05) | 0.15  (0.06) | 0.26 (0.08) | 0.54  (0.18) | 1.06  (0.31) | 2.31  (0.92) | 2.68  (1.04) | - |  |
|  | **Tmax (Hr)** |  |  |  |  |  |  |  |  |  |
|  | n | 3 | 3 | 4 | 10 | 15 | 22 | 9 | 0 |  |
|  | Median | 3 | 2 | 2.52 | 2.01 | 2.15 | 3 | 3 | - |  |
|  | (Range) | 2-3 | 2-3 | 2-3 | 2-4 | 1.05-4 | 1.98-8 | 2-3.02 | - |  |
|  | **Tlast (Hr)** |  |  |  |  |  |  |  |  |  |
|  | n | 3 | 3 | 4 | 7 | 15 | 20 | 9 | 0 |  |
|  | Median | 168 | 168 | 168 | 167 | 167 | 167 | 168 | - |  |
|  | (Range) | 166-169 | 168-215 | 168-168 | 165-167 | 165-192 | 164-216 | 120-189 | - |  |
|  | **AUC(0-tlast) (Hr*ug/mL)** |  |  |  |  |  |  |  |  |  |
|  | n | 3 | 3 | 4 | 7 | 15 | 19 | 9 | 0 |  |
|  | Mean  (SD) | 6.2  (1.53) | 8.77  (4.37) | 12.1  (4.01) | 22.6  (8.81) | 51.8  (14.3) | 99.8  (43.2) | 137  (53.5) | - |  |
|  | **T1/2 (Hr)** |  |  |  |  |  |  |  |  |  |
|  | n | 2 | 1 | 2 | 5 | 11 | 13 | 4 | 0 |  |
|  | Median | 287 | 209 | 127 | 174 | 182 | 183 | 294 | - |  |
|  | (Range) | 163-412 | 209-209 | 122-133 | 151-259 | 73.4-813 | 22.6-3850 | 98.3-409 | - |  |
| **Cycle 1**  **Day 1** | **Cmax (ug/mL)** |  |  |  |  |  |  |  |  |  |
|  | n | 3 | 3 | 4 | 10 | 15 | 22 | 9 | 10 |  |
|  | Mean  (SD) | 0.14 (0.06) | 0.17  (0.02) | 0.276 (0.0695) | 0.5  (0.14) | 1.07  (0.27) | 2.25  (1.15) | 2.72  (0.69) | 1.13  (0.23) |  |
|  | **Tmax (Hr)** |  |  |  |  |  |  |  |  |  |
|  | n | 3 | 3 | 4 | 10 | 15 | 22 | 9 | 10 |  |
|  | Median | 4 | 3 | 3.53 | 3 | 2 | 2.58 | 3 | 22.3 |  |
|  | (Range) | 2-4 | 2-3.08 | 1.92-6.08 | 2-6 | 0-4 | 1-4 | 1-4.02 | 2-25.2 |  |
|  | **Tlast (Hr)** |  |  |  |  |  |  |  |  |  |
|  | n | 3 | 3 | 4 | 9 | 14 | 21 | 8 | 10 |  |
|  | Median | 25 | 22.9 | 23.8 | 24.4 | 24 | 24.1 | 24 | 23.8 |  |
|  | (Range) | 21.5-27.1 | 21.5-24.4 | 23.3-24.3 | 23.4-26 | 23-26.3 | 21.1-24.6 | 23.1-25.6 | 21.5-25.2 |  |
|  | **AUC(0-tlast) (Hr*ug/mL)** |  |  |  |  |  |  |  |  |  |
|  | n | 3 | 3 | 4 | 9 | 14 | 21 | 8 | 10 |  |
|  | Mean  (SD) | 2.08  (0.61) | 2.65  (0.41) | 3.93  (0.68) | 7.28  (1.67) | 15.7  (4.01) | 28.1  (11.6) | 39  (10.1) | 18.4  (3.7) |  |
| **Cycle 1**  **Day 15** | **Cmax (ug/mL)** |  |  |  |  |  |  |  |  |  |
|  | n | 3 | 3 | 4 | 8 | 15 | 19 | 9 | 9 |  |
|  | Mean  (SD) | 0.13 (0.04) | 0.12  (0.03) | 0.2 (0.02) | 0.42  (0.16) | 0.85  (0.23) | 1.65  (0.63) | 2.65  (1.1) | 1.83  (0.65) |  |
|  | **Tmax (Hr)** |  |  |  |  |  |  |  |  |  |
|  | n | 3 | 3 | 4 | 8 | 15 | 19 | 9 | 9 |  |
|  | Median | 3 | 3.07 | 2.52 | 5.13 | 2.75 | 3.93 | 4 | 3 |  |
|  | (Range) | 2.58-4 | 2.12-4 | 1-4 | 2-25 | 0.967-4.5 | 1-6.08 | 0-22.1 | 1-23.3 |  |
|  | **Tlast (Hr)** |  |  |  |  |  |  |  |  |  |
|  | n | 3 | 3 | 4 | 7 | 14 | 16 | 9 | 9 |  |
|  | Median | 25.3 | 22.5 | 24.1 | 24 | 24 | 23.7 | 23.4 | 23.8 |  |
|  | (Range) | 22.9-28 | 22.4-23.5 | 23.4-24.8 | 22.8-25.4 | 18.9-28.9 | 18.3-25.3 | 19.8-24.1 | 21.6-24.2 |  |
|  | **AUC(0-tlast) (Hr*ug/mL)** |  |  |  |  |  |  |  |  |  |
|  | n | 3 | 3 | 4 | 7 | 14 | 16 | 9 | 9 |  |
|  | Mean  (SD) | 2.67  (0.6) | 2.3  (0.78) | 4.15  (0.34) | 8.59  (3.4) | 16.1  (5) | 28.5  (11.7) | 46.8  (23) | 35.4  (13.7) |  |
| **Steady**  **State**** | Cmin (ug/mL) |  |  |  |  |  |  |  |  |  |
|  | n | 3 | 3 | 3 | 6 | 12 | 18 | 6 | 8 |  |
|  | Mean  (SD) | 1.23  (0.22) | 1.51  (0.82) | 1.82  (0.28) | 4.31  (1.22) | 7.31  (1.99) | 13.1  (5.57) | 21.8  (3.76) | 15.5  (7.47) |  |
| * First dose is the PK run-in dose in Arm 2. | | | | | | | | | | |
| ** Steady state: Cmin is calculated based on the average of trough concentrations from Cycle 2 Day 1 and onwards. | | | | | | | | | | |

**Appendix table 3: Immunohistochemical analysis of cytoplasmic biomarkers.**

| **Tissue Biomar- ker**** |  | **DLs 1 - 4**  **(N=20)** | **DL 5**  **N=15** | **DL 6**  **N=23** | **DLs 7 and 7.1 N=19** | **All patients N=77** |
| --- | --- | --- | --- | --- | --- | --- |
| **pMEK** | n | 11 | 2 | 6 | 4 | 23 |
|  | Mean (SD) | +0.8 (44.4) | 0 | -16.1 (17.3) | -2.6 (5.3) | -4.3 (32) |
|  | Median | -11.1 | 0 | -15.3 | 0 | -9.1 |
|  | Min-Max | -38.1 - +122.2 | 0 | -40.0 - +4.0 | -10.5 - 0 | -40.0 - +122.2 |
| **pERK** | n | 2 | 2 | 5 | 4 | 13 |
|  | Mean (SD) | +52.3 (80) | +27.1 (56) | -27.0 (40.9) | -17.8 (11.5) | -3.6 (48.6) |
|  | Median | +52.3 | +27.1 | -50.00 | -13.5 | -11.8 |
|  | Min-Max | -4.0 - +109.1 | -12.5 - +66.7 | -63.5 - +19.5 | -34.6 - -9.5 | -63.5 - +109.1 |
| **BIM** | n | 8 | 1 | 2 | 3 | 14 |
|  | Mean (SD) | -18 (65.1) | -100 | -7.1 (11.1) | +6.7 (5.8) | -17.00 (54.5) |
|  | Median | -14.3 | -100 | -7.1 | +10 | 0 |
|  | Min-Max | -100 - +100 | -100 - -100 | -15.0 - +0.7 | 0 - +10.0 | -100 - +100 |
| **CKIT** | n | 4 | 0 | 1 | 3 | 8 |
|  | Mean (SD) | -60 (80) |  | 0 | 0 | -30. (61.4) |
|  | Median | -100 |  | 0 | 0 | 0 |
|  | Min-Max | -100 - +60.0 |  | 0 | 0 | -100 - +60.0 |
| **PAKT473** | n | 11 | 1 | 4 | 3 | 19 |
|  | Mean (SD) | +20.1(132.9) | -4.6 | -48.4(55.9) | 0 | +1.2 (105.4) |
|  | Median | -20 | -4.6 | -46.7 | 0 | -4.6 |
|  | Min-Max | -100 - +400.0 | -4.6 - -4.6 | -100 - 0 | 0 | -100 - +400 |
| **PS6** | n | 8 | 1 | 6 | 4 | 19 |
|  | Mean (SD) | -16.8 (20.9) | +255.6 | -20.6 (29.1) | 33.4 (55.7) | +6.9 (70.8) |
|  | Median | -18.9 | +255.6 | -35.8 | +16.3 | -13 |
|  | Min-Max | -53.3 - +12.5 | +255.6 - +255.6 | -43.6 - 29.6 | -13.0 - +114.3 | -53.3 - +255.6 |
| **PTEN** | n | 8 | 2 | 5 | 3 | 18 |
|  | Mean (SD) | -5 (44.1) | 0 | -9.4 (27) | 10 (17.3) | -3.16 (32.4) |
|  | Median | -7.4 | 0 | 0 | 0 | 0 |
|  | Min-Max | -72.0 - +83.3 | 0 | -57.1 - 10 | 0 - 30 | -72.0 - +83.3 |

**Appendix Table 5: Immunohistochemical analysis of nuclear biomarkers.**

| **Tissue Biomar- ker**** |  | **DLs 1 - 4 N=20** | **DL 5**  **N=15** | **DL 6**  **N=23** | **DLs 7 and 7.1 N=19** | **All patients N=77** |
| --- | --- | --- | --- | --- | --- | --- |
| **pERK** | n | 10 | 2 | 5 | 4 | 21 |
|  | Mean (SD) | +21.1 (52.13) | +18.2 (35.8) | -41.8 (48) | -20.3 (31.3) | -2 (51.6) |
|  | Median | 0 | +18.2 | -22.2 | -7.6 | 0 |
|  | Min-Max | -17.5 - +135.7 | -7.1 - +43.5 | -93.7 - 0 | -66.1 - 0 | -93.7 - +135.7 |
| **Ki67** | n | 11 | 2 | 6 | 4 | 23 |
|  | Mean (SD) | +0.21 (59) | 0 | -47.93 (40.8) | -6.08 (20) | -13.46 (50) |
|  | Median | -11.1 | 0 | -51.8 | -2.6 | -11.1 |
|  | Min-Max | -95.7 - +150.0 | 0 | -95.7 - +20.0 | -33.3 - +14.3 | -95.7 - +150.0 |
| **PARP** | n | 8 | 0 | 3 | 3 | 14 |
|  | Mean (SD) | +65.1 (180.5) |  | +20.00 (121.2) | +222.2 (167.8) | +89.1 (172.3) |
|  | Median | -20 |  | 0.00 | +200.00 | +33.3 |
|  | Min-Max | -100 - +400.0 |  | -90.0 - +150.0 | +66.7 - +400.0 | -100 - +400.0 |
| **Cyclin D1** | n | 10 | 3 | 5 | 5 | 23 |
|  | Mean (SD) | -4.5 (95) | +56.8 (125.6) | -49.12 (30.7) | +25 (113.6) | +0.19 (93.7) |
|  | Median | -24 | +5.00 | -48.9 | 0 | -25 |
|  | Min-Max | -100 - +233.3 | -34.6 - +200.0 | -77.8 - 0 | -71.4 - +221.4 | -100 - +233.3 |
| **MITF** | n | 9 | 2 | 6 | 4 | 21 |
|  | Mean(SD) | +400.6(1312.5) | +11.8 (23.7) | +20.1 (111.3) | +98.6 (239.7) | +197.3(856.9) |
|  | Median | -27.3 | +11.8 | -6.60 | 0 | -13.2 |
|  | Min-Max | -80.0 - 3900 | -5.0 - 28.6 | -75.0 - 240.0 | -61.1 - +455.6 | -80 - +3900 |
| **P27** | n | 9 | 2 | 5 | 4 | 20 |
|  | Mean (SD) | -2.2 (44) | -40 (56.6) | +156.4 (312.2) | +261.1 (427.3) | +86 (251.9) |
|  | Median | 0 | -40 | +30.4 | +72.2 | +5 |
|  | Min-Max | -50 - +72.7 | -80.0 - 0 | -73.7 - +700 | 0 - +900.0 | -80.0 - +900.0 |
| **P53** | n | 10 | 2 | 4 | 4 | 20 |
|  | Mean (SD) | -27.30 (49.484) | +82.33(190.5) | +49.32 (123.273) | +188.5 (400.62) | +42.14 (195.033) |
|  | Median | -29.2 | +82.4 | -3.8 | 0 | +-3.8 |
|  | Min-Max | -100 - +70.0 | -52.4 - +217.1 | -28.6 - +233.3 | -35.0 - +788.9 | -100 - +788.9 |
|  | | | | | | |
